# MicroRNA-34a: A Versatile Regulator of Myriads of Targets in Different Cancers

**DOI:** 10.3390/ijms18102089

**Published:** 2017-10-02

**Authors:** Ammad Ahmad Farooqi, Sobia Tabassum, Aamir Ahmad

**Affiliations:** 1 Laboratory for Translational Oncology and Personalized Medicine, Rashid Latif Medical College, Lahore 54000, Pakistan; ammadfarooqi@rlmclahore.com; 2 Department of Bioinformatics and Biotechnology, International Islamic University, Islamabad 44000, Pakistan; sobia.tabasum@iiu.edu.pk; 3 Department of Oncologic Sciences, Mitchell Cancer Institute, University of South Alabama, Mobile, AL 36604, USA

**Keywords:** miR-34a, non-coding RNA, tumor-suppressor

## Abstract

MicroRNA-34a (miR-34a) is a tumor suppressor that has attracted considerable attention in recent years. It modulates cancer cell invasion, metastasis, and drug resistance, and has also been evaluated as a diagnostic and/or prognostic biomarker. A number of targets of miR-34a have been identified, including some other non-coding RNAs, and it is believed that the modulation of these myriads of targets underlines the versatile role of miR-34a in cancer progression and pathogenesis. Seemingly appealing results from preclinical studies have advocated the testing of miR-34a in clinical trials. However, the results obtained are not very encouraging and there is a need to re-interpret how miR-34a behaves in a context dependent manner in different cancers. In this review, we have attempted to summarize the most recent evidence related to the regulation of different genes and non-coding RNAs by miR-34a and the advances in the field of nanotechnology for the targeted delivery of miR-34a-based therapeutics and mimics. With the emergence of data that contradicts miR-34a’s tumor suppressive function, it is important to understand miR-34a’s precise functioning, with the aim to establish its role in personalized medicine and to apply this knowledge for the identification of individual patients that are likely to benefit from miR-34a-based therapy.

## 1. Introduction

MicroRNA-34a (miR-34a) has attracted overwhelming interest in last several years because of its ability to modulate myriad of oncogenic functions in different cancers [1,2,3,4,5,6,7]. Not only has its role been demonstrated in cancer metastasis [8,9] and drug resistance [10], it is now being evaluated as a diagnostic as well as a prognostic biomarker [11,12,13]. As a direct impact of this interest, a number of reviews have been written about the regulation of different pathways by miR-34a in various cancers, preclinical studies related to miR-34a mimics, and different nanotechnological strategies to improve the delivery of miR-34a to the target cells, in last few years [14,15,16,17]. However, our knowledge about this miRNA is rapidly evolving and this review discusses the most recent research on miR-34a, with a focus on highlighting the research findings from the last two years.

## 2. Activation and Expression of miR-34a

Different mechanisms have been proposed for the activation of miR-34a. There are some new and exciting pieces of evidence that reveal that mature miR-34 is present in an inactive state in the cells and lacks a 5′-phosphate [18]. However, DNA-damage triggers the activation of miR-34 mainly through 5′-end phosphorylation in an ATM (Ataxia-telangiectasia Mutated Kinase)- and Clp1 (Cleavage and Polyadenylation Factor I Subunit-1)-dependent manner that enables loading into Argonaute 2. Essentially, activation of miR-34 through this pathway occurs quickly and does not require de-novo p53-modulated transcriptional regulation [18]. Surprisingly, miR-34a activation, following DNA damage, was independent of p53 [18]. Data clearly indicates the rapid response of cells to DNA damage, with a pre-existing transcribed pool of miR-34a, which can be rapidly activated via phosphorylation.

DDX3X (DEAD-box RNA helicase) interacts with the Drosha/DGCR8 complex and substantially enhances the processing activity of Drosha/DGCR8 complex on pri-miRNAs with increased mature miRNA expression [19]. DDX3X-dependent pri-miRNA-34a significantly interacts with DDX3X. However, DDX3X inhibition strictly impairs binding of pri-miRNA-34a to DDX3X. Hence, it was experimentally verified that DDX3X promotes the biogenesis of different microRNAs [19]. BRCA1 (breast cancer 1) is also involved in speeding up the processing of miRNA primary transcripts [20]. BRCA1 was found to increase the expression levels of both precursor and mature forms of miR-34a, miR-16-1, and miR-145. Additionally, BRCA1 interacted directly with DDX5 and DROSHA of the DROSHA micro-processor complex, and it interacted with SMAD3 (Mothers Against Decapentaplegic), DHX9 (DEAH Box Polypeptide-9) RNA helicase and p53 [20]. BRCA1 recognized RNA secondary structures and interacted with miRNA primary transcripts through a DNA-binding domain [20]. It was suggested that BRCA1 regulated biogenesis of miRNAs mainly through the DROSHA micro-processor complex and SMAD3/p53/DHX9 pathway.

**Figure 1:**
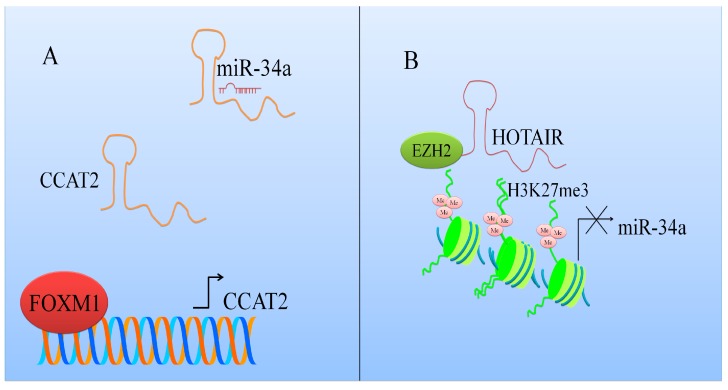
Schematic description of how different long non-coding RNAs regulate MicroRNA-34a (miR-34a) mediated activities. (**A**) CCAT2 interacts with miR-34a [22] and protects oncogenic mRNAs from degradation; (**B**) HOX Transcript Antisense RNA (HOTAIR) repressed the expression of miR-34a.

miR-34a was reported to be considerably enhanced in DZnep (3-Deazaneplanocin A)-treated SW1990 and PANC1 cells [21]. Enhancer of Zeste (EZH2) was noted to transcriptionally repress miR-34a in pancreatic ductal adenocarcinoma (PDAC) cells. miR-34a was found to be upregulated in EZH2 depleted PDAC cells. Tumor growth was significantly delayed in nude mice that were xenografted with EZH2 silenced SW1990 cells. It was shown that HOX Transcript Antisense RNA (HOTAIR) mediated miR-34a inhibition by EZH2 (Figure 1). Furthermore, HOTAIR knockdown resulted in an increase in miR-34a expression in EZH2 overexpressing cancer cells. High enrichment of HOTAIR was observed at promoter region of miR-34a. HOTAIR inhibition markedly reduced H3K27me3 (tri-methylation of lysine 27 on histone H3) levels and EZH2 occupancy at the promoter region of miR-34a [21].

It has been reported that NF-κB p65 subunit overexpression induced an increase in the levels of miR-34a in esophageal squamous EC109 cancer cells, whereas ectopic expression of dominant negative IkappaB significantly reduced the expression of miR-34a [23]. An interesting role of p53 was established in NF-κB-mediated miR-34a transcriptional activity as only wild type p53 was found to be responsible for this NF-κB effect. In esophageal cancer cell lines with mutant or decreased p53, NF-κB could still bind to the promoter of miR-34a, but its overexpression did not result in induced miR-34a expression. This points to a complex regulation of miR-34a expression that needs to be elucidated further.

miR-34a has also been reported to be downregulated in different cancers. For example, miR-34a expression was low in serum and intra-tumoral tissues and noted to be associated with an increased incidence of bone metastasis in hepatocellular carcinoma (HCC) patients [9]. Low levels of miR-34a in intra-tumoral tissues increased vascular invasion in HCC patients. The rate of overall metastases was higher in HCC patients with lower expression of miR-34a [9].

## 3. miR-34a-Mediated Regulation of Different Targets

miRNAs function through the regulation of their multiple targets. This is true for miR-34a as well, and multiple studies have documented the effects of miR-34a on its various targets. Here, we discuss the effects of miR-34a on its target proteins and lncRNAs (long non-coding RNAs), as reported within the last two years (Table 1). miR-34a has been shown to promote apoptotic cell death and inhibit autophagy by directly targeting HMGB1 (High Mobility Group Box-1) in AML cells [24]. Also, the overexpression of miR-34a significantly inhibited the migratory and invasive potential of esophageal squamous cell carcinoma (ESCC) cells via inhibition of FNDC3B (Fibronectin Type III Domain Containing 3B), MMP2 (matrix metalloproteinase-2) and MMP9 expression levels [25]. IGF2BP3 (Insulin-like growth factor-2 mRNA-binding protein 3) overexpression has been reported to be correlated with poorer survival in gastric cancer patients [26]. IGF2BP3 was directly targeted by miR-34a in MKN28 and AGS cells. Re-expression of IGF2BP3 partially impaired miR-34a mediated anti-carcinogenic effects [26].

**Figure 2:**
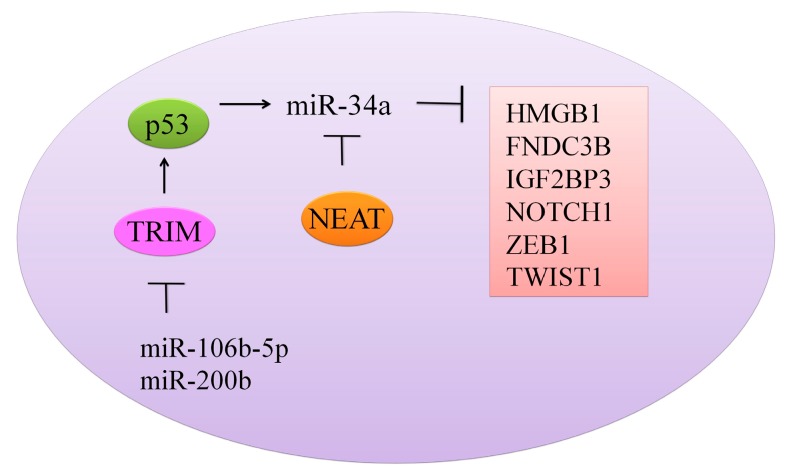
Different oncogenes and proteins regulated by miR-34a.

TRIM8 (Tripartite motif containing protein-8), a tumor suppressor protein is noted to be directly targeted by miRNA-106b-5p and miRNA-17-5p [29]. miRNA-17-5p or miRNA-106b-5p suppression by antagomirs induced an increase in the expression of TRIM8 with notably reduced cell proliferation. miRNA-17-5p and miRNA-106b-5p overexpression inhibited TRIM8, which consequently resulted in the destabilization of p53 and N-MYC oncogene activation. There was a formation of a feed-forward loop whereby N-MYC promoted its own protein expression mainly through TRIM8 inhibition by promoting miRNA-106b-5p and miRNA-17-5p expression. However, miR-17-5p and miR-106b-5p inhibition induced an increase in the level of TRIM8, enhanced p53 stability, and activated miR-34a, which in turn repressed theN-MYC protein (Figure 2) [29].

With the growing interest in non-coding RNAs in cancer research [32], the effect of miR-34a on these RNAs has also been evaluated. Nuclear paraspeckle assembly transcript-1 (NEAT1), a recently reported lncRNA, is involved in regulation of gene expression by retention of mRNAs for editing in the nucleus [28] (Figure 2). NEAT1 knockdown resulted in arrest of cells in the G0/G1 phase. Overexpression of NEAT1 induced downregulation of miR-34a in renal cell carcinoma (RCC) cells [28]. Linc-ROR (long intergenic non-protein coding RNA, regulator of reprogramming) consists of 4 exons, and it is located at chromosome 18q21.31 [27]. It plays a crucial role in the regulation of cellular reprogramming. Inhibition of linc-ROR decreased cell viability and induced apoptotic cell death in gemcitabine-treated breast cancer MDA-MB-231 cells. Different pathways have been documented to coordinate autophagosomal assembly at the molecular level, and intriguingly, Linc-ROR inhibition promoted autophagosomal assembly in gemcitabine-treated MDA-MB-231 cells. Chromatin immunoprecipitation was used to study histone H3 acetylation in the promoter region of miRNA-34a. miRNA-34a promoter H3 acetylation was dramatically reduced in gemcitabine treated groups. Inhibition of linc-ROR inhibited histone H3 acetylation in promoter region of miR-34a and reduced its expression [27].

A single miRNA can regulate multiple target genes while, at the same time, a single gene can be targeted by multiple miRNAs [33]. It has been suggested, through studies that involved in silico analysis, that miR-34a can bind to target sequences of five members of protein kinase-C family (PRKCA, PRKCB, PRKCE, PRKCQ and PRKCH) [34]. The investigators went on to validate these bioinformatics predictions by transfecting miR-34a into HEK 293T and Jurkat cells, which resulted in a reduction in the protein levels of PKC isozymes PRKCA, PRKCB, and PRKCQ, thus demonstrating a biological validation of predicted miR-34a-target interactions.

## 4. miR-34a Co-Operates with Multiple microRNAs to Target Different Genes

As discussed above, miR-34a functions through the regulation of its multiple targets. Through its targets, miR-34a modulates a diverse range of biological functions [35,36,37,38,39,40,41] (Table 2). In addition to studies that have evaluated the role of miR-34a alone in cancer pathogenesis, there are some reports that have evaluated the functional involvement of multiple miRNAs in cancer progression and/or therapy, with miR-34a being a part of the miRNA panel. For example, ST3 Beta-Galactoside Alpha-2,3-Sialyltransferase 5 (ST3GAL5), a sialyltransferase gene, has been shown to be directly targeted by miR-34a, miR-548l, and miR-26a [42]. Overexpression of miR-34a, miR-548l, and miR-26a in MHCC97-L or MHCC97-H cells substantially changed their malignant behaviors and oncogenicity. The size and weight of tumors were notably reduced in mice inoculated with miR-34a, miR-548l, and miR-26a, and miR mixture mimics groups [42]. Data clearly suggested that miR-34a in combination with different tumor suppressor miRNAs negatively regulated ST3GAL5 to inhibit hepatocellular carcinoma.

Fucosyltransferase is involved in the transfer of an l-fucose sugar from a GDP-fucose donor substrate to an acceptor substrate and has recently been reported to be correlated with cancer progression [43]. miRNA-34a and miRNA-26a targeted FUT8 (fucosyltransferase 8) in HCC cells. The enforced expression of miRNA-34a or miRNA-26a considerably downregulated FUT8 at both the protein and mRNA level in MHCC97H cells. Moreover, inhibition of miRNA-34a or miRNA-26a upregulated FUT8 in MHCC97L cells. This suggests that miR-34a and miR-26a are involved in the negative regulation of mRNA stability of FUT8. Subcutaneous implantation of miRNA mimics-transfected MHCC97H cells into the right flank of nude mice was carried out to study the in vivo effects. One week later, the mice were given intratumoral injections of either miR mimics or mimic control, 3-times/week, for the duration of 3 weeks. Significant regressions in tumor weight and volume were noticed in miRNA-34a or miRNA-26a mimics-expressing xenografts, as compared to the controls. Furthermore, miRNA-34a and miRNA-26a markedly inhibited FUT8 and proliferation-associated marker Ki67 in the tumor tissues [43].

## 5. miR-34’s Role in Cancer Treatment

The potential role of miR-34a in cancer therapy has also been tested in various pre-clinical studies. Combination of doxorubicin with sorafenib or miRNA-34a or sorafenib and miRNA-34a synergistically suppressed the proliferation of osteosarcoma 143B cells. Synergy of triple-drug combinations was evident at varying concentrations [44]. 80% inhibition of osteosarcoma cells required approximately 160 nM doxorubicin alone, 25 nM miRNA-34a alone, or 10,000 nM sorafenib alone, however, only 40 nM doxorubicin, 4 nM miRNA-34a, and 4000 nM sorafenib in the triple-drug combination were sufficient to produce similar results. Triple-drug therapy effectively suppressed the growth of the tumor and spontaneous pulmonary metastases in mouse models. The number of metastases identified, per lung sample, were considerably lower in animal groups treated with triple-combination. The sum of the metastatic tumor diameters in groups treated with triple-combination and doxorubicin/miRNA-34a-treated group was significantly smaller [44].

0404, a DNA-damaging compound, concentration-dependently, upregulated miR-34a in HepG2 cells (p53 wild-type) [45]. SIRT1 protein expression was downregulated with the increasing concentrations of 0404, while the levels of acylated p53 were upregulated with the increasing concentrations of 0404. 4 μmol/kg of 0404 significantly reduced tumor growth in mice xenografted with HepG2 cells. p53 was upregulated in the HepG2 tumors excised from 0404 treated nude mice [45]. On the other hand, oxaliplatin was reported to downregulate miR-34a and upregulate TGF-β (transforming growth factor β)/Smad4 in colorectal cancer patients [46]. miR-34a expression was downregulated but TGF-β and Smad4 mRNA levels increased significantly in the blood samples of colorectal cancer patients after chemotherapy. miR-34a was downregulated in HT29 and HT29-OXA cells upon exposure to 10 μmol/L oxaliplatin for 48 h, although the HT29-OXA cells expressed lower levels of miR-34a as compared to HT29 cells with or without oxaliplatin. Smad4 is directly targeted by miR-34a in some types of tumor cells. HT29-OXA cells expressed higher TGF-β levels, which consequently resulted in Smad4 upregulation. miR-34a mimics downregulated expression levels of TGF-β and Smad4 in HT29 and HT29-OXA cells [46].

Low-frequency magnetic fields (LF-MFs) have been shown to be effective against lung cancer. LF-MF exposure induced significant inhibition of tumor growth in mice inoculated subcutaneously with lewis lung cancer cells [47]. Mechanistically, LF-MF exposure triggered the upregulation of miR-34a to inhibit lung cancer growth. E2F3 and E2F1 levels were notably reduced in the cells that ectopically expressed miR-34a. Likewise, LF-MF exposure downregulated E2F3 and E2F1 protein levels in LLC cells. Significant reduction in the levels of E2F3 and E2F1 was observed in tumor tissue of LF-MF exposed xenografted mice. Data demonstrated that LF-MF exposure effectively inhibited lung cancer through miR-34a upregulation and the downregulation of E2F pathway in lung cancer [47]. Further, second and third generation EGFR-tyrosine-kinase-inhibitors (TKIs) in combination with miR-34a were effective against cells having secondary erlotinib resistance [48].

MUC1, a heterodimeric oncoprotein is frequently overexpressed in AML. PD-L1 expression was markedly suppressed in MUC1 silenced THP-1 and MOLM-14 cells [49]. MUC1 inhibition markedly increased miR-200c and miR-34a levels without exerting any effect on precursor microRNA in AML cells. Significantly higher levels of miR-34a and miR-200c were observed in MUC1 silenced THP-1 and MOLM-14 cells [49]. It has recently been reported that miR-34a overexpression in multiple myeloma (MM) cells and MM-CSCs decreased lytic bone lesions and tumorigenicity in NOD/SCID mice (Non-obese diabetic/severe combined immunodeficiency) [50]. The size of the tumors was significantly smaller in miR-34a-CD138-CD34-CSCs group (1109 mm^3^ ± 76 mm^3^). Bone mineral density was considerably enhanced in mice injected with miR-34a-MM CSCs [50]. miRNA-34a is frequently downregulated in diffuse malignant peritoneal mesothelioma (DMPM) [51]. DMPM cells reconstituted with miR-34a had significantly reduced proliferation potential and tumorigenicity. miR-34a-mimics-transfected cells were subcutaneously (MesoII, STO, MP8) and intraperitoneally (MP8, STO) inoculated into SCID mice. miR-34a impaired tumor growth in all of the subcutaneously xenografted mice, with maximum tumor inhibitory effects ranging from 57 to 98%. Moreover, an appreciably delayed tumor onset was recorded in MP8 and MesoII cell models [51].

Epithelial to mesenchymal transition (EMT) is a complex mechanism intricately modulated by different extracellular signals and several activating transcription factors. mRNA levels of NOTCH1, ZEB1, and TWIST1 were noted to be significantly reduced in BT-549 cells transfected with miR-34a mimics [52]. EMT is also linked to cancer stem cell (CSC) phenotype [53]. HOTAIR (Hox transcript antisense intergenic RNA) was found to be frequently overexpressed in CSC-MB231 and CSC-MCF7 populations [54]. HOTAIR transcriptionally repressed miR-34a and enhanced colony formation, proliferation, invasion, and self-renewal capacity of breast CSCs [54]. It is becoming systematically more understandable that body responds immunologically to nucleic acid drugs by “switching on” different mechanisms that included RIG-I-like receptors (RLRs), Toll-like receptors (TLRs) and double-stranded RNA (dsRNA)-activated protein kinase (PKR) [55]. Therefore, to avoid any immunological response, 30-nucleotides based single-stranded RNA was designed and termed as “guide hairpin RNA (ghRNA, ghR)”. Structurally, the lack of passenger strand seed sequences reduced unwanted repression of genes related to existing short RNA reagents. Tumor regression was noted in xenografted mice systemically and locally injected with ghR-form miR-34a (ghR-34a) [55].

## 6. Multi-Layered Complexity of Competitive Endogenous RNAs Crosstalk and Competition with miR-34a

Competitive endogenous RNA (ceRNA) hypothesis suggests that transcripts with shared microRNA binding sites compete for posttranscriptional control [56,57]. lncARSR (lncRNA Activated in RCC with sunitinib resistance) was reported to be overexpressed in renal cell carcinoma (RCC) cells resistant to sunitinib [58]. Based on the insights from previously reported research [59,60,61], it is clear that cell-secreted exosomes undergo internalization by neighboring cells. Intracellularly increased levels of lncARSR were noticed upon incubation with exosomes from resistant cells. However, this accumulation was not observed in lncARSR-knockdown-resistant cells [58]. It was experimentally verified that lncARSR was transferred to recipient-cells mainly through exosomes. lncARSR was localized in cytosol and behaved as a ceRNA to sequester miRNAs which consequently resulted in the release of corresponding miRNA-targeted transcripts. Functional miRNA-449 and miRNA-34a binding sites were present in lncARSR. Repression of miRNA-34a and miRNA-449 with miRNA-34a sponges restored sunitinib resistance in lncARSR-silenced cells. On the contrary, miR-34a mimic transfection significantly reduced lncARSR-induced sunitinib resistance. Intriguingly, lncARSR behaved as a molecular sponge for miRNA-449 and miRNA-34 and facilitated AXL and c-MET expression [58].

Taurine upregulated 1 (TUG1), a lncRNA is a frequently overexpressed in different cancers [31]. miRNA-299 and miRNA-34a significantly reduced VEGFA and lncRNA-TUG1 in endometrial cancer cells. VEGFA (Vascular endothelial growth factor) was markedly reduced in lncRNA-TUG1 knockdown cells in presence of miR-34a and miR-299. These observations suggest that lncRNA-TUG1 works as a ceRNA and regulated levels of VEGFA by sponging miRNAs [31].

## 7. Nanotechnological Strategies to Deliver miR-34a

With the growing realization of the potential role of miR-34a in cancer therapy, efforts are under-way to optimize strategies for the targeted delivery of this miRNA. Cell-penetrating peptides PepFect (PF)14 have been proposed as efficient carriers for the delivery of miR-34a into primary prostate carcinoma-1 (PPC-1) cells [62]. Lipid-based transfection reagents (siPORT, LF2000, and RNAiMAX) have also been tested to identify agents with the highest efficiency of delivery. Although RNAiMAX and LF2000 efficiently delivered miRNA, they also had pronounced negative effects on cell viability, because of the associated toxicity. There was a relatively weaker effect of siPORT-transfected miR-34a on target gene expression. miR-34a transfection using siPORT also did not influence cell viability, which suggested that siPORT had low to no associated toxicity, but, at a high cell density optimal for LF2000, siPORT had relatively lower transfection efficiency [62].

ATP (Adenosine-Triphosphate)—responsive delivery system has been shown to be an efficient strategy to deliver cargo to the target cells [63]. In line with this approach, ATP aptamer and its cDNA sequence were hybridized for generation of duplex, into which doxorubicin (DOX) interacted through the GC-rich motif of duplex. PEI25K (polyethyleneimine (PEI) with a molar mass of 25 kD) was used as a carrier to condense the DOX-loading duplex and miR-34a for the construction of ternary nano-complex PEI/DOX-Duplex/miR-34a [63]. Chondroitin sulfate-functionalized polyamidoamine dendrimers (PAMAM) have also been used to deliver miR-34a [64]. CS-PAMAM/miR-34a considerably reduced tumor growth in xenografted mice [64], thus, documenting some benefits of this approach.

Polymeric nanogels (NG) are also being tested for efficacy to deliver the payload to target cells. Nanogels are hydrogel particles (nano-sized), having three-dimensional networks composed of hydrophilic swellable polymers. Expectedly, the average tumor volume of the NG3/miR-34a treated group showed significant tumor regression (379 ± 175 mm^3^) as compared to NG3/NC-miR (883 ± 580 mm^3^) (Shatsberg et al, 2016).

## 8. Natural Products-Mediated Regulation of miR-34a

Recent years have also witnessed a renewed interest in the cancer chemopreventive and/or cancer therapeutic ability of natural products, the “nutraceuticals” [65]. One reason for this interest is the documented pleiotropic activity of these anticancer agents. Additionally, many of these compounds have also been demonstrated to modulate miRNA-expression and function, leading to their anticancer effects [66]. One such nutraceutical, thymoquinone, an active molecule isolated from *Nigella sativa*, was recently reported to be effective against breast cancer cells. Thymoquinone, in combination with miR-34a, markedly reduced the mRNA levels of NOTCH1, ZEB1, and TWIST1 in treated breast cancer cells [52]. Thus, co-administration of miR-34a and thymoquinone could effectively reverse EMT. Since EMT has been implicated in cancer metastasis and resistance to therapy, this is an exciting observation that needs to be further tested in pre-clinical models. Further, AU-1, a spirostanol saponin present in Agavaceae plants, was noted to inhibit SIRT1 (silent expression regulator-1), an NAD-dependent deacetylase [30]. AU-1 upregulated miR-34 to effectively reduce SIRT1 levels in renal adenocarcinoma ACHN cells. SIRT1-knockdown induced transient but significantly increased p21/Cip1 protein levels [30]. Please check and confirm.

## 9. Darker Side of miR-34a in Different Cancers

Most of the published reports have established a tumor-suppressive function of miR-34a. Therefore, its over-expression has mostly been shown to reduce cancer cells’ aggressiveness, suggesting its delivery to tumors as a therapeutic strategy. However, some studies have reported a contrasting function of miR-34a. Delta-like ligand 1 (DLL1) was noted to sensitize osteosarcoma cells to different drugs [67]. miR-34a mimic transfection in osteosarcoma G-292 cell line partially increased the resistance against drugs, with resistance against cisplatin increased 1.66-folds. Moreover, miR-34a antagomiR transfection in SJSA-1 cell line reduced chemoresistance against different drugs. DLL1 expression was downregulated in xenografted mice intratumorally injected with miR-34a antagomiR-transfected G-292 cells. In contrast, DLL1 expression increased significantly in tumors of mice injected with miR-34a antagomiR transfected SJSA-1 cell line [67]. Therefore, this data revealed that miRNA-34a made osteosarcoma cells multi-chemoresistant, mainly through the downregulation of DLL1.

Kaposi’s sarcoma-associated herpesvirus (KSHV)/human herpesvirus 8 (HHV-8) is a DNA tumor virus [68]. It encodes different homologues of genes, including G protein-coupled receptor vGPCR. This receptor is homologically similar to the human chemokine receptors CXCR1 and CXCR2. Constitutive activation of vGPCR was noticed in the ligand un-bound state. vGPCR structurally interacted with a number of CC and CXC chemokines, and induced KS-like lesions in mice when transgenically expressed. Viral encoded GPCR induced repression of genomic stability associated pathways in vGPCR-TC#1 cells mainly through inhibition of miR-34a target genes. MiR-34a was noted to promote genomic instability, and the targeted inhibition of miR-34a resulted in an increase in the expression level of the genes associated with genomic stability [68]. Further, the overexpression of miR-34a, miR-155, and miR-200c was noted to correlate positively with colorectal cancer [69]. Reciprocal overexpression of the miR-34 family has previously been documented in chromosomal areas with frequent allelic loss. miR-34a knockdown significantly exerted inhibitory effects on the proliferation potential of not only renal carcinoma cells, but also of MCF7 and HeLa cells [70]. In light of these reports, it is critical to fully understand the functional role of miR-34a, particularly its cancer-specific function, through more mechanistic and detailed studies.

## 10. Clinical Trials Involving miR-34a

Because of the promising results with miR-34a in experimental and pre-clinical studies, it has also been tested in clinical settings. MRX34, a liposomal miR-34a mimic was given intravenously 2 days/week to HCC patients [71]. MRX34 with dexamethasone pre-medication had acceptable safety and demonstrated anti-tumor response in subset of patients with advanced refractory solid tumors. No pre-medications were given in the initial two dosage levels of 10 mg/m^2^ and 20 mg/m^2^. Maximally tolerated dosage was 110 mg/m^2^ for non-HCC and 93 mg/m^2^ for HCC patients [71]. Mirna Therapeutics, Inc. (Nasdaq:MIRN), a clinical stage biopharmaceutical company recently closed clinical study in which MRX34 was used in cancer patients to utilize the potential of miR-34a in the treatment of cancer (https://clinicaltrials.gov/ct2/show/NCT01829971). Questions were raised about clinical efficacy of MRX34 when multiple immune-related severe adverse events (SAE) were recorded in patients administered with MRX34 during the clinical trial.

## 11. Conclusions

miR-34a has emerged as a promising tumor-suppressive miRNA. Based on the overwhelmingly increasing scientific evidence it is clear that miR-34a inhibits tumor growth via modulation of an array of oncogenes. Its in vitro anticancer potential has been validated in multiple in vivo mice models. This has led to efforts targeting its delivery to cancer cells, for potential therapeutic interventions. miR-34a mimics have been tested in combination with different chemotherapeutic drugs to maximize the targeted killing of cancer cells, and the effective reduction in tumor burden. Novel strategies to nanotechnologically deliver miR-34a have also shown some potential. However, a few recent reports have suggested the tumor-promoting role of miR-34a, which should not be ignored. Infact, such adverse effects of miR-34a need to be critically evaluated. It is possible that the effects of miR-34a are cancer specific and only patients with some specific cancers will benefit from miR-34a therapy. In this age of precision and personalized medicine, it needs to be clearly established as to which subset of cancer patients are more likely to benefit from miR-34a therapy.

## Figures and Tables

**Table 1:** Targets of miR-34a ^1^.

Target	Target Type	Cancer	Reference
FNDC3B (Fibronectin Type III Domain Containing 3B)	Gene	Esophageal	[25]
HMGB1 (High Mobility Group Box-1)	Gene	Acute Myeloid Leukemia	[24]
IGF2BP3 (Insulin-like growth factor-2 mRNA-binding protein 3)	Gene	Gastric	[26]
Linc-ROR (long intergenic non-protein coding RNA, regulator of reprogramming)	lncRNA	Breast	[27]
MMP2 (matrix metalloproteinase-2)	Gene	Esophageal	[25]
NEAT1 (Nuclear paraspeckle assembly transcript-1)	lncRNA	Renal	[28]
N-MYC (basic helix-loop-helix protein 37)	Gene	Renal/colorectal	[29]
SIRT1 (silent expression regulator-1)	Gene	Renal	[30]
TUG1 (Taurine upregulated 1)	lncRNA	Endometrial	[31]

^1^ This review focuses on reports from last two years only.

**Table 2:** Biological Functions affected by miR-34a targets ^1^.

Target	Mechanism	Biological Function	References
DAPK2 (Death-associated protein kinase 2), Sp1 (specificity protein 1)	miR-34a inhibits apoptosis of dendritic cells through repression of DAPK2/Sp1 pathway	Immunotherapy	[35]
AR (Androgen Receptor)	Negative regulation of AR by miR-34a increases ULBP2 expression and enhances NK cell activity	Immunotherapy	[36]
PD-L1 (Programmed death-ligand 1)	miR-34a can modulate PD-L1, an important mediator of immune response	Immunotherapy	[37]
VEGFA (Vascular endothelial growth factor A)	LncRNA-TUG1 functions as a miR-34a sponge in hepatoblastoma cells, and TUG1-miR-34a-VEGFA axis affects angiogenesis	Angiogenesis	[31]
CD44	Reduced blood vessel density observed in tumors treated with miR-34a	Angiogenesis	[38]
Bcl-2	miR-34a promotes apoptosis	Apotosis	[39,40]
Sirtuin-1 (SIRT-1)	SIRT-1 is negatively regulated by miR-34a	Apoptosis	[41]

^1^ This review focuses on reports from last two years only. LncRNA: long non coding RNA, TUG1: taurine upregulated 1.

## Abbreviations

ATM	Ataxia-telangiectasia Mutated Kinase
ATP	Adenosine-Triphosphate
ceRNA	Competitive endogenous RNA
Clp1	Cleavage and Polyadenylation Factor I Subunit-1
CSC	Cancer stem cell
DDX3X	DEAD-box RNA helicase
DHX9	DEAH Box Polypeptide-9
DLL1	Delta-like ligand 1
DMPM	Diffuse malignant peritoneal mesothelioma
dsRNA	Double-stranded RNA
DZnep	3-Deazaneplanocin A
EMT	Epithelial to mesenchymal transition
ESCC	Esophageal squamous cell carcinoma
FNDC3B	Fibronectin Type III Domain Containing 3B
ghRNA	Guide hairpin RNA
HCC	Hepatocellular carcinoma
HHV-8	Human herpesvirus 8
HMGB1	High Mobility Group Box-1
HOTAIR	Hox transcript antisense intergenic RNA
IGF2BP3	Insulin-like growth factor-2 mRNA-binding protein 3
KSHV	Kaposi’s sarcoma-associated herpesvirus
LF-MFs	Low-frequency magnetic fields
Linc-ROR	Long intergenic non-protein coding RNA, regulator of reprogramming
LncARSR	lncRNA Activated in RCC with sunitinib resistance
LncRNAs	long non-coding RNAs
NEAT1	Nuclear paraspeckle assembly transcript-1
PDAC	Pancreatic ductal adenocarcinoma
PKR	Protein kinase (PKR)
PPC-1	Primary prostate carcinoma-1
RLR	RIG-I-like receptor
SIRT1	Silent expression regulator-1
SMAD3	Mothers Against Decapentaplegic
ST3GAL5	ST3 Beta-Galactoside Alpha-2,3-Sialyltransferase 5
TKIs	Tyrosine-kinase-inhibitors
TLR	Toll-like receptor
TRIM8	Tripartite motif containing protein-8
TUG1	Taurine upregulated 1
VEGFA	Vascular endothelial growth factor-A
